# Kinetic Theory of Self-Propelled Particles with Nematic Alignment

**DOI:** 10.3390/e26121054

**Published:** 2024-12-04

**Authors:** Horst-Holger Boltz, Benjamin Kohler, Thomas Ihle

**Affiliations:** Institute for Physics, University of Greifswald, 17489 Greifswald, Germany

**Keywords:** dry active matter, kinetic theory, active nematics, Vicsek-like models

## Abstract

We present the results from kinetic theory for a system of self-propelled particles with alignment interactions of higher-order symmetry, particularly nematic ones. To this end, we use the Landau equation approach, a systematic approximation to the BBGKY hierarchy for small effective couplings. Our calculations are presented in a pedagogical way with the explicit goal of serving as a tutorial from a physicists’ perspective into applying kinetic theory ideas beyond mean-field to active matter systems with essentially no prerequisites and yield predictions without free parameters that are in quantitative agreement with direct agent-based simulations

## 1. Introduction

The seminal work on the Vicsek model [1,2] showing a phase transition to a state with long-ranged order in a two-dimensional model of inter-aligning self-propelled particles, a transition which would be in opposition to the Mermin–Wagner theorem [3] for equilibrium systems, can reasonably be used to mark the beginning of an ongoing foray from a theoretical perspective into understanding the “new” physics in models of active matter. In general, active matter is a subfield of nonequilibrium systems that are driven out of equilibrium on the smallest scale considered. The prototypical realization of this are self-propelled particles, as in the case of the Vicsek model. In a real-world version of such a system, there will be some microscopic mechanism (the usage of fuel, the metabolism of a living creature, external driving forces, etc.) that falls into the broader class of nonequilibrium physics, but if taken as fundamental units, these self-propelled particles are *active*.

In the decades since the first publication of the Vicsek model, considerable progress has been made in active matter physics, both on an analytical–computational level [4,5,6,7,8] and in the controlled experimental realization [9,10] of active matter systems. A somewhat comprehensive list of related review articles can be found in ref. [11]. The status of Vicsek-like models (an alternative nomenclature is that of *dry active aligning matter* [8]) as a workhorse in the research, particularly for more fundamental insights, has prevailed. This is not only limited to the original aim of studying flocking [12], the onset of collective motion or a global *polar* order in the directions of motion, but also extends to exploring systems with anti-aligning behavior [13,14,15,16,17,18,19], as well as systems with higher-order symmetry.

The most direct extension from polar order is the case of nematic order. Active nematics [20,21,22,23,24,25,26,27,28,29] are of high practical relevance, as many abundant instances of active-matter systems, such as bacteria and polymers, are based on particles with one distinct axis (rod-shaped). Typically, the alignment interaction in these systems is the result of actual collisions (in the sense that forces are acting on the spatial degrees of freedom) and the resulting symmetry is less clear, as inelastic collisions can generally induce a polar order [17,30,31]. Here, we consider the case of microscopically nematic pure alignment interactions. In line with the active matter concept, this can be seen as an abstraction to more microscopic interactions, such as steric interactions or by other means, say hydrodynamically [32].

On the technical side, interest in kinetic approaches to statistical physics has been renewed in the context of active matter [15,18,33,34,35,36,37,38,39,40,41,42,43,44,45,46,47,48,49,50]. The extension of maximum entropy concepts (a discussion with some historical notes regarding the different conceptual schools can, for example, be found in ref. [51]) to these systems is not straightforward, whereas kinetic theory is systematically built up starting from the equations of motion and can therefore be adapted to a great variety of problems with few underlying assumptions. Specific Langevin dynamics being given, a systematic marginalization of the corresponding Fokker–Planck equation establishes the Born–Bogoliubov–Green–Kirkwood–Yvon (BBGKY) hierarchy of *n*-particle functions. Most physical quantities of interest can be written in terms of the one-particle function, making this a natural starting point. Closure of the hierarchy has been discussed in the literature [42,45] on polar models in detail. Often employed is the assumption of molecular chaos, which leads to mean-field dynamics (which can also be derived as the formal N→∞ limit of the equations of motion [35,52,53,54,55,56]) which have been referred to as the Vlasov (or McKean–Vlasov [52]) equation [57,58] (see also the earlier work by Jeans [59] and ref. [60] for an argument to call it the *collisionless* Boltzmann equation).The emergence of mean-field dynamics in certain continuum limits is the subject of active mathematical research, often being referenced as the propagation of chaos [43,52,61,62,63,64]. A full treatment of dynamics *beyond mean-field* is intricate and usually limited to perturbative approaches in either the density or the coupling strength. The density route, neglecting contributions from non-pair collisions, leads to the proper *Boltzmann equation*, which is valid for arbitrary interaction strengths [65]. As there are several technical intricacies related to this method (see the discussion of polar models in ref. [15]), we follow the route of weak interactions here. This leads [66] to the *Landau equation*, which arguably could also be named after Bogoliubov and/or Balescu, who provided more careful systematic derivations [67,68,69]. In particular, it becomes apparent under diligent derivation that the Landau equation is valid at arbitrary densities for sufficiently small coupling (the limit of higher densities, but weak interaction does only exist in models with soft interactions; relevant steric interactions, for example, are not suitable for a perturbative treatment in interaction strength). The Landau equation has also seen usage in the case of polar models [46,70]. There exist rigorous statements that the spatially homogeneous Landau equation that we will derive is indeed the proper limit of the Boltzmann equation with weak interactions [63,71,72].

The bottom-up nature of this kind of kinetic theory is complementary to more top-down mesoscopic hydrodynamic approaches that try to formulate the effective equations of motion for slow, collective variables from general principles [12,36,43,73,74,75].

In this work, we present some (in parts rather old) ideas on kinetic theory, particularly the Landau equation approach, in a concise and self-contained way that is tailored to the (rather new in the grand scheme of things) application in Vicsek-like models and, along the way, we derive novel results for Vicsek-like systems with nematic (and generally higher-order symmetry) coupling.

## 2. Model

**Figure 1:**
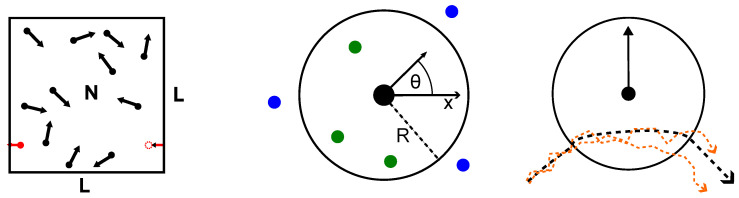
Schematic explanation of the model used and the relevant control parameters. We consider ensembles of *N* self-propelled agents moving in two dimensions within a quadratic box of size *L* with periodic boundary conditions applied in each direction. Together, *N* and *L* fix the (number) density $\rho =N/L^{2}$. Adjacency for interaction is determined via an interaction radius *R*, and the interaction takes place with other agents within this radius (marked green in the middle panel as opposed to those marked blue that are outside). The interaction will change the orientational angle θ that encodes the direction of flight of the particle. It is measured with respect to a fixed reference axis, in our case the *x*-axis. The size of the radius *R* fixes the dimensionless density $M=\rho \pi R^{2}$, the expected number of particles within one interaction zone. Particles propagate with constant velocity $v_{0}$. Without any interactions, the direction of flight changes only subject to noise (orange trajectories in the right panel, depicted in the resting frame of an interaction partner) whose strength is given by $D_{r}$. Upon interaction, the rate of change is controlled by the interaction strength Γ. Throughout the manuscript, we use non-dimensional equations giving rise to a non-dimensional interaction strength $\mathrm{Sc}=\Gamma R/v_{0}$ and an inverse diffusion constant (Peclet number) $\mathrm{Pe}=v_{0}/\left(RD_{r}\right)$.

We consider a class of generalized Vicsek-like models [34,35] wherein particles are described by two-dimensional positions $x_{i}$ (i=1,…,N) and orientational angles $\theta_{i}$ whose dynamics are given by

(1a) $$\begin{array}{cc} \dot{x_{i}} & =v_{0}{\hat{n}}_{i} \end{array}$$

(1b) $$\begin{array}{cc} \dot{\theta_{i}} & =\Gamma \;\sum_{j\in \Omega_{i}}\sin\left(n\left(\theta_{j}\left(t\right)-\theta_{i}\left(t\right)\right)\right)+\eta_{i} \end{array}$$

with $\hat{n_{i}}={\left(\cos\theta_{i},\sin\theta_{i}\right)}^{T}$, $\langle \eta_{i}\rangle =0$ and $\langle \eta_{i}\left(t\right)\eta_{j}\left(t^{\prime }\right)\rangle =2D_{r}\delta_{i,j}\delta \left(t-t^{\prime }\right)$. It is possible to formulate analogous dynamics in higher spatial dimensions [35,76]. Many aspects of our discussion are model-independent, but we limit the discussion to the two-dimensional case as the simplest non-trivial realization of orientational interactions whenever necessary. A schematic of our model is depicted in Figure 1. Crucially, this specific model does not include a prefactor that scales the interaction according to the number of interaction agents. This is essential in the limit of many interacting partners, as will be discussed below.

Thus, we consider ensembles of self-propelled particles that move with a fixed propulsion speed $v_{0}$ in the plane along the direction given by their orientational angle (relative to some fixed axis). These angles change via purely orientational or *alignment* interactions, whose strength is given by Γ, and via explicit noise, whose strength is given by the resulting rotational diffusion constant $D_{r}$. Crucially, the interaction topology changes over time as the set of particles $\Omega_{i}$ considered adjacent to particle *i* change. Specifically, throughout this work, we use

(1c) $$\begin{array}{c} \Omega_{i}=\left\{k\neq i|\left|x_{i}-x_{k}\right|\leq R\right\}, \end{array}$$

i.e., every particle has an identical fixed *interaction radius R* and interacts with every particle within a circle of radius *R* around it. For specific applications, other models of adjacency, such as metric-free models [39,77,78,79], can be more appropriate.

The interaction radius introduces a relevant length scale into the system, which gives rise to a suitable control parameter of the system, the non-dimensional density $M=\pi R^{2}\rho =\pi R^{2}N/L^{2}$. The intuitive meaning of this is that there are on average about *M* particles within the interaction radius of one particle. Similarly, we define the non-dimensional interaction strength $\mathrm{Sc}=\Gamma R/v_{0}$ and the non-dimensional inverse diffusion strength or Peclet number $\mathrm{Pe}=v_{0}/\left(RD_{r}\right)$. This work focuses on the case of weakly interacting particles (to be specified later on) without any requirement regarding diluteness (i.e., arbitrary *M*).

**Figure 2:**
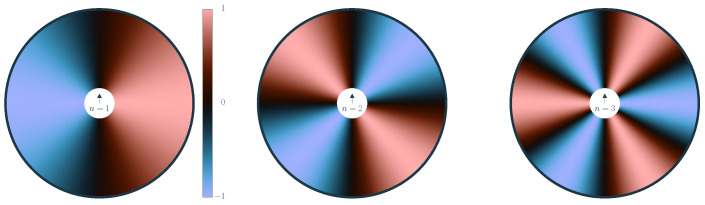
Visualization of the action of the *chelation number n* within the model of the interaction. The color map shows the relative interaction with a second particle exerted by a pivot particle moving upwards (shown in the middle) as a function of the orientation of the second particle (the polar angle). A positive value indicates that a counter-clockwise turn is induced. Equilibrium orientations are marked in black. For a positive Γ, the angles that lie in a transition from red to blue in a counter-clockwise orientation are stable, while for a negative Γ, the other zeroes are stable. The case of polar interaction corresponds to n=1, whereas n=2 represents nematic interactions. The symmetry of the interaction is also apparent in simulation snapshots in the ordered phase (see Figure 3).

**Figure 3:**
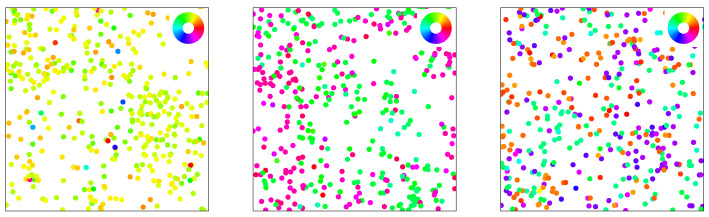
Snapshots of agent-based simulations in the respective ordered phase (positive coupling Γ) for (from left to right) chelation numbers n=1,2,3. Agents are represented by a circle whose size is given by the interaction radius *R*. Their orientation angles $\theta_{i}$ are indicated via a color code (see inserted wheel). The correspondence to the symmetry of the interaction itself, cp. Figure 2, is apparent as the states show polar order for n=1 with a collective motion of particles in one direction, nematic ordering for n=2 where all particles move along one axis in both directions, and a collective state with three-fold symmetry (green/turquoise, orange, and violet/blue) for n=3.

Finally, the kind of interaction between any pair of angles is crucially affected by the *chelation number* n∈N in the argument of the interaction kernel. This allows us to control the symmetry of the alignment interaction, cf. Figure 2, with n=1 corresponding to the case of polar interactions and n=2 to nematic interactions. Also shown is the case of triatic interactions for n=3 as a representative for higher *n*-atic interaction (as observed, for example, in dihedral liquid crystals [80]) types with *n*-fold symmetry. A suitable order parameter is given by the bond order parameter $m_{l}=\sum_{i}e^{il\theta_{i}}$ [81]. Generally, there are *n* stable and *n* unstable equilibria. While we are focused on n=2 and the case of n=1 has been treated [46,70] in the literature, we provide a general treatment for the sake of self-containedness. The symmetry of the interaction kernel directly affects the positional statistics observed in agent-based simulations (to be introduced in Section 4), see Figure 3.

## 3. Landau Kinetic Theory

By means of standard stochastic calculus [82,83], we find the Fokker–Planck equation for the full *N*-particle distribution function $P_{N}$ to the Langevin Equation (1) after rescaling to units such that $R=v_{0}=1$,

(2) $$\begin{array}{cc} \partial_{t}P_{N}\left(\left\{x_{i},\theta_{i}\right\}\right) & =-\sum_{i=1}^{N}\left(\left({\hat{n}}_{i}\cdot \nabla_{i}\right)P_{N}+\mathrm{Sc}\partial_{\theta_{i}}\left(\sum_{j=1}^{N}a_{i,j}\sin\left(n\left(\theta_{j}-\theta_{i}\right)\right)P_{N}\right)-{\mathrm{Pe}}^{\;-1}\partial_{\theta_{i}}^{2}P_{N}\right) \end{array}$$

wherein the omitted arguments of $P_{N}$ are the same on the right as on the left, which we will use throughout to increase legibility, and we introduce an indicator function

(3) $$\begin{array}{cc} a_{i,j} & =\Theta (1-\left|x_{i}-x_{j}\right|). \end{array}$$

The distribution $P_{N}$ contains the full information for all 3N microscopic degrees of freedom, which makes it unwieldy to use. As we consider all the particles to be identical, most relevant quantities, in particular the order parameter, should be reducible to integrals over a *one-particle* distribution. Therefore, we follow the textbook [69,84,85,86,87,88] Born–Bogoliubov–Green–Kirkwood–Yvon (BBGKY) hierarchy [67,89,90,91] approach and introduce reduced *k*-particle distribution functions by integrating out all the degrees of freedom except for those of the first *k* particles,

(4) $$\begin{array}{cc} p_{k} & =\frac{N!}{(N-k)!}{\left(\frac{1}{M}\right)}^{k}\int \ldots \int P_{N}\;dx_{k+1}\;\;d\;\theta_{x+1}\ldots \;\;d\;x_{N}\;\;d\;\theta_{N}. \end{array}$$

The combinatorial pre-factor accounts for possible other selections of the *k* pivot particles. The non-standard pre-factor ${\left(L^{2}/\left(\pi R^{2}N\right)\right)}^{k}={\left(1/M\right)}^{k}$ is chosen such that all $p_{k}$ are of the order O(1) for a uniform distribution $P_{N}$. Using the shorthand notation $i={\left(x_{i},\theta_{i}\right)}^{T}$, the evolution of the one-particle distribution, i.e., the first member of the BBGKY hierarchy, is given by

(5a) $$\begin{array}{c} \partial_{t}p_{1}\left(1,t\right)=\left(L_{1}+{\mathrm{Pe}}^{-1}\partial_{\theta_{1}}^{2}\right)p_{1}\left(1,t\right)-\gamma \;\partial_{\theta_{1}}\;\int \int \;a_{1,2}\sin\left(n\left(\theta_{2}-\theta_{1}\right)\right)p_{2}\left(1,2,t\right)\;\;d\;2. \end{array}$$

Here, we introduce the notation γ=ScM for the effective interaction strength, the parameter that controls applicability of the theory, and make use of the one-particle Liouville operator $L_{i}=-{\hat{n}}_{i}\nabla_{i}$ determining the evolution in the free deterministic ($\mathrm{Sc}={\mathrm{Pe}}^{-1}=0$) problem. As for any system with additive pair interactions, the evolution of the *k*-particle function relies on the k+1-particle function establishing the BBGKY hierarchy. In particular, the two-particle function is found to evolve as

(5b) $$\begin{array}{c} \begin{array}{cc} \; & \partial_{t}p_{2}\left(1,2,t\right)=\left(L_{1}+L_{2}+{\mathrm{Pe}}^{-1}\left(\partial_{\theta_{1}}^{2}+\partial_{\theta_{2}}^{2}\right)\right)p_{2}\\ \; & -\gamma \;[M^{-1}\left(\partial_{\theta_{1}}\left(a_{1,2}\sin\left(n\left(\theta_{2}-\theta_{1}\right)\right)p_{2}\right)+\partial_{\theta_{2}}\left(a_{2,1}\sin\left(n\left(\theta_{1}-\theta_{2}\right)\right)\right)p_{2}\right)\\ \; & +\partial_{\theta_{1}}\;\;\;\int \int \;\;\;a_{1,3}\sin\left(n\left(\theta_{3}-\theta_{1}\right)\right)p_{3}\left(1,2,3,t\right)\;\;d\;3+\partial_{\theta_{2}}\;\;\;\int \int \;\;\;a_{2,3}\sin\left(n\left(\theta_{3}-\theta_{2}\right)\right)p_{3}\left(1,2,3,t\right)\;\;d\;3]. \end{array} \end{array}$$

The (abridged) hierarchy of Equation (5) is the starting point for any meaningful kinetic theory. Before moving on conceptually, it is very advantageous to separate off some contributions to the higher-order functions. In the free problem, all the particles are uncorrelated and one therefore has factorizing *k*-particle functions $p_{k,\mathrm{free}}=\prod_{i=1^{k}}p_{1}\left(i,t\right)$. In light of this, we perform an *Ursell* (or *Mayer* or *cluster*) expansion [92,93] introducing *reduced correlation functions* $g_{i}$. The correlation function $g_{k}$ contains the novel contributions on the *k*-particle level such that it contains the information about $f_{k}$ that is not contained in the lower-order $f_{j}$ and $g_{j}$ with j<k. In particular, the first three correlation functions are (omitting time dependencies for clarity here and in the following) given by

(6) $$\begin{array}{c} \begin{array}{cc} p_{1}\left(1\right) & =g_{1}\left(1\right)\\ p_{2}\left(1,2\right) & =p_{1}\left(1\right)p_{1}\left(2\right)+g_{2}\left(1,2\right)\\ p_{3}\left(1,2,3\right) & =p_{1}\left(1\right)p_{1}\left(2\right)p_{1}\left(3\right)+p_{1}\left(1\right)g_{2}\left(2,3\right)+p_{1}\left(2\right)g_{2}\left(1,3\right)+p_{1}\left(3\right)g_{2}\left(1,2\right)+g_{3}\left(1,2,3\right). \end{array} \end{array}$$

**Figure 4:**
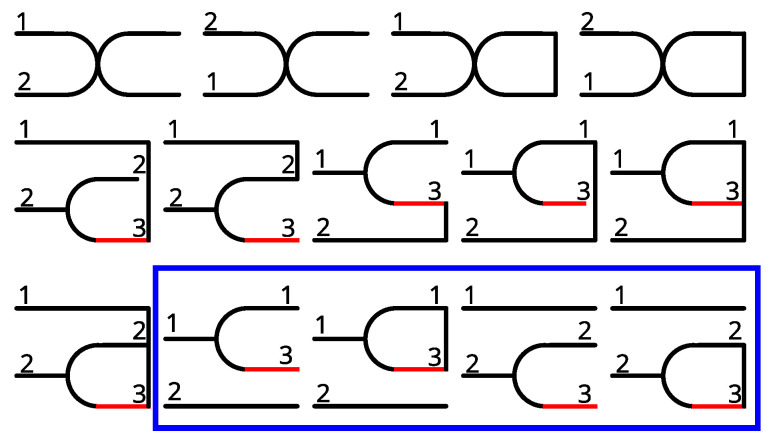
Diagrammatic representation, as explained in the main text, of the terms on the two-particle level, Equation (5b), after performing the expansion of Equation (6). Only fully connected diagrams contribute to the evolution of $g_{2}$. We indicate the not fully connected diagrams conveying trivial information that is already separated off in the Ursell form by a blue box. These do not contribute to $g_{2}$. To enhance clarity and differing from ref. [69], we mark virtual indices (corresponding to the degrees of freedom that are to be integrated over) by red lines and employ a convention that the derivatives occurring at vertices (junctions of three or more lines) are to be taken with respect to the uppermost index coming from the left. The free propagators are not explicitly represented graphically.

We follow the diagrammatic approach of Balescu [69] to reduce the resulting equations for $\partial_{t}g_{2}$ to terms that actually contribute, utilizing a kinetic equivalent of the linked cluster theorem. We represent reduced one-particle distribution functions by unconnected horizontal lines that are tagged by their respective particle index; a *k*-tuple of horizontal lines connected by a vertical line represents a reduced *k*-particle correlation function. Each diagram contains one vertex that corresponds to the interaction kernel, $∼\gamma \partial_{\theta }\sin\left(n\left(\theta^{\prime }-\theta \right)\right)$ (with a convention that the derivative is to be taken with respect to the (upper, where ambiguous) incoming line) and, finally, the lines corresponding to the given degrees of freedom start on the left and all other degrees of freedom (red lines in the figure) are to be integrated over. The important part to understand is that only fully connected (in the sense that every point on any line can be reached on lines from any other point) diagrams can contribute to the correlation function. In Figure 4, we show all the diagrams corresponding to the result of naively inserting Equation (6) into Equation (5b). The diagrams contained within the blue box are not fully connected and, therefore, do not contribute to $g_{2}$. Unaffected by these considerations is the free (Sc=0) part of the time evolution. Reconstituting these considerations into equations, we find that

(7) $$\begin{array}{c} \begin{array}{cc} \; & \left(\partial_{t}-F_{1,2}\right)g_{2}\left(1,2\right)=-\gamma [M^{-1}\{\partial_{\theta_{1}}\left(a_{1,2}\sin\left(n\left(\theta_{2}-\theta_{1}\right)\right)\right)\left(p_{1}\left(1\right)p_{1}\left(2\right)+g_{2}\right)\\ \; & +\partial_{\theta_{2}}\left(a_{2,1}\sin\left(n\left(\theta_{1}-\theta_{2}\right)\right)\right)\left(p_{1}\left(1\right)p_{1}\left(2\right)+g_{2}\right)\}\\ \; & +\partial_{\theta_{1}}\;\int \int \;a_{1,3}\sin\left(n\left(\theta_{3}-\theta_{1}\right)\right)\left(p_{1}\left(1\right)g_{2}\left(2,3\right)+p_{1}\left(3\right)g_{2}+g_{3}\left(1,2,3\right)\right)\;\;d\;3\\ \; & +\partial_{\theta_{2}}\;\int \int \;a_{2,3}\sin\left(n\left(\theta_{3}-\theta_{2}\right)\right)\left(p_{1}\left(2\right)g_{2}\left(1,3\right)+p_{1}\left(3\right)g_{2}+g_{3}\left(1,2,3\right)\right)\;\;d\;3] \end{array} \end{array}$$

wherein $F_{1,2}=\left(L_{1}+L_{2}+{\mathrm{Pe}}^{\;-1}\left(\partial_{\theta_{1}}^{2}+\partial_{\theta_{2}}^{2}\right)\right)$ is the free two-particle Fokker–Planck operator. As stated earlier, we are interested in the weakly interacting limit, which in light of the resulting equations, we can specify to be γ=ScM≪1. To this end, there is a fruitful insight to be drawn from the fact that only fully connected diagrams contribute to the $g_{k}$: any fully connected *k*-particle diagram will have to have one vertex and at least k−2 lines linked vertically by correlation functions, meaning that we can conclude that $g_{k}=O\left({\mathrm{Sc}}^{k-1}\right)$. Thus, all of the hierarchical terms in any $g_{k}$ evolution, i.e., those originally belonging to $p_{k+1}$-terms and containing integrals, are of a higher order in γ. To order $O({\mathrm{Sc}}^{2})$, we therefore obtain the following Landau kinetic theory

(8a) $$\begin{array}{cc} \partial_{t}p_{1}\left(1\right) & =\left(L_{1}+{\mathrm{Pe}}^{\;-1}\partial_{\theta_{1}}^{2}\right)p_{1}-\gamma \;\partial_{\theta_{1}}\;\;\;\int \int \;\;\;a_{1,2}\sin\left(n\left(\theta_{2}-\theta_{1}\right)\right)\left(p_{1}p_{1}\left(2\right)+g_{2}\left(1,2\right)\right)\;\;d\;2\\ \; & \equiv \left(L_{1}+{\mathrm{Pe}}^{\;-1}\partial_{\theta_{1}}^{2}\right)p_{1}-J_{\mathrm{coll}} \end{array}$$

(8b) $$\begin{array}{cc} \partial_{t}g_{2}\left(1,2\right) & =F_{1,2}\;g_{2}\left(1,2\right)+Q\left(1,2\right) \end{array}$$

with the source term (using $a_{1,2}=a_{2,1}$)

(8c) $$\begin{array}{cc} Q(1,2) & =-\mathrm{Sc}\;a_{1,2}\left(\partial_{\theta_{1}}-\partial_{\theta_{2}}\right)\sin\left(n\left(\theta_{2}-\theta_{1}\right)\right)p_{1}\left(1\right)p_{2}\left(2\right). \end{array}$$

It is a great strength of this approach that it is directly apparent that the *collision integral* $J_{\mathrm{coll}}=J_{\mathrm{coll}}\left(1\right)$ consists of two contributions, a mean-field one, $J_{\mathrm{coll}}^{\mathrm{MF}}$, which is obtained by neglecting correlations and is of the order O(γ), and a genuinely Landau contribution, $J_{\mathrm{coll}}^{L}$, of the order $O(M{\mathrm{Sc}}^{2})$ for which we have to know $g_{2}$ *during interactions* by solving Equation (8b). That is, we split

(9a) $$\begin{array}{cc} J_{\mathrm{coll}} & =J_{\mathrm{coll}}^{\mathrm{MF}}+J_{\mathrm{coll}}^{L} \end{array}$$

(9b) $$\begin{array}{cc} J_{\mathrm{coll}}^{\mathrm{MF}} & =\gamma \;\partial_{\theta_{1}}\;\int \int \;a_{1,2}\sin\left(n\left(\theta_{2}-\theta_{1}\right)\right)p_{1}p_{1}\left(2\right)\;\;d\;2 \end{array}$$

(9c) $$\begin{array}{cc} J_{\mathrm{coll}}^{L} & =\gamma \;\partial_{\theta_{1}}\;\int \int \;a_{1,2}\sin\left(n\left(\theta_{2}-\theta_{1}\right)\right)g_{2}\left(1,2\right)\;\;d\;2. \end{array}$$

Enslaving $g_{2}$ to $p_{1}$, i.e., pretending we know $p_{1}\left(1\right)$ and $p_{1}\left(2\right)$ as functions of time, we can directly give this solution formally as a variation of constants

(10) $$\begin{array}{c} g_{2}\left(1,2,t\right)=U_{1,2}\left(t_{0},t,{\mathrm{Pe}}^{\;-1}\right)g_{2}\left(1,2,t_{0}\right)+U_{1,2}\left(t_{0},t,{\mathrm{Pe}}^{\;-1}\right)\int_{t_{0}}^{t}U_{1,2}^{-1}\left(t_{0},t^{\prime },{\mathrm{Pe}}^{\;-1}\right)Q\left(t^{\prime }\right)\;\;d\;t^{\prime } \end{array}$$

with the *free two-particle propagator*

(11) $$\begin{array}{c} U_{1,2}\left(t_{0},t,{\mathrm{Pe}}^{\;-1}\right)=e^{F_{1,2}\left(t-t_{0}\right)}. \end{array}$$

Operating in the weak noise limit, we set $U_{1,2}\left(t_{0},t,{\mathrm{Pe}}^{\;-1}\right)\approx U_{1,2}\left(t_{0},t,0\right)$, neglecting noise contributions during the (typically short) timescale of interactions but keeping the diffusive terms arising from the (typically large) timescale of free flight. Additionally, the one-particle operators trivially commute and the system is symmetric under translation in time, so that we find that

(12) $$\begin{array}{cc} U_{1,2}\left(t_{0},t,{\mathrm{Pe}}^{\;-1}\right) & \approx U_{1,2}\left(t_{0},t,0\right)=e^{\left(L_{1}+L_{2}\right)\left(t-t_{0}\right)}=U_{1}\left(t_{0},t\right)U_{2}\left(t_{0},t\right). \end{array}$$

Recalling the definition of the one-particle Liouville operator, $L_{i}=-{\hat{n}}_{i}\nabla_{i}$, which is simply the generator of infinitesimal translations, we see that the time propagation mediated by $U_{1,2}$ is indeed a displacement by $\Delta x=n_{i}\Delta t$. This is the physical expectation: in Landau kinetic theory, we compute the contributions from interactions assuming that particles move freely during the interaction, which is free ballistic transport in this problem. With this in mind, the back propagation mediated by $U^{-1}\left(t_{0},t\right)=U_{1,2}\left(t,t_{0}\right)$ is just a translation to the location where the agents had been at an earlier time.

To progress, two *physically informed* approximations are necessary. Firstly, we have to specify the meaning of the time $t_{0}$ and the value of the correlation function at this time. As stated previously, we only have good control over the propagator on short timescales and, by construction, the correlation function $g_{2}\left(1,2,t\right)$ only contains information if particles 1 and 2 have interacted. More importantly, as these particles subsequently interact with other (screened out) particles, any built-up correlation will decrease over time. This leads to the natural choice of assuming *one-sided* molecular chaos: we treat particles as uncorrelated at the beginning of a pair interaction [84]. The distinction of one-sidedness is important here, as the molecular chaos assumption (originally coined as the Boltzmann property by Kac [61]) usually refers to a mean-field description, i.e., $g_{2}\equiv 0$ throughout. Mathematically, these considerations provide $t_{0}$ with the meaning of the onset of an interaction (i.e., the point in time when the two particles enter each other’s interaction radii) and we can impose $g_{2}\left(1,2,t_{0}\right)=0$ as the particles being assumed to be uncorrelated at this point in time. We will address $t_{0}$ later on.

As an aside, we use physics terminology here, wherein mean-field is used to describe the effective dynamics derived from a neglection of correlations. It is an ongoing effort within the mathematics literature to show that the correlations are vanishing and that the long-term *N*-particle probability distribution functions factorize when considering the direct continuum (N→∞) limit of Equation (1). This, again following Kac [61], is referred to as the *propagation of chaos* [55,62,64,94]. For Vicsek-type models, this is discussed in ref. [55]. While we do omit contributions O(1/N) (from the combinatorial pre-factors), our perspective is that *N* is finite throughout (most ensembles of active matter particles such as flocks of birds or schools of fish are tiny, perhaps $10^{2–4}$, compared to the typical particle numbers considered in, say, gases, which are of the order of moles, so $10^{23}$), and if at all, the formal limit N→∞ should be performed at the end for actual observables such that the control parameters M,Pe,Sc remain finite.

Secondly, we know that in the relevant limits, ${\mathrm{Pe}}^{\;-1},\gamma \ll 1$, the one-particle distributions are spatially homogeneous on the scales of interactions reducing the action of the propagator solely to that which it has on the indicator function $a_{1,2}$, which evaluates to one during the interaction. In summation, we can write Equation (10) as

(13) $$\begin{array}{cc} g_{2}\left(1,2,t\right) & =-\mathrm{Sc}\;\int_{t_{0}}^{t}U_{1,2}\left(t^{\prime },t\right)a_{1,2}\left(\partial_{\theta_{1}}-\partial_{\theta_{2}}\right)\sin\left(n\left(\theta_{2}-\theta_{1}\right)\right)p_{1}\left(1,t\right)p_{1}\left(2,t\right)\;dt^{\prime }\\ & =-\mathrm{Sc}\;\left(\partial_{\theta_{1}}-\partial_{\theta_{2}}\right)\sin\left(n\left(\theta_{2}-\theta_{1}\right)\right)p_{1}\left(1,t\right)p_{1}\left(2,t\right)\int_{t_{0}}^{t}U_{1,2}\left(t^{\prime },t\right)a_{1,2}\;dt^{\prime }\\ & =-\mathrm{Sc}\;\left(\partial_{\theta_{1}}-\partial_{\theta_{2}}\right)\sin\left(n\left(\theta_{2}-\theta_{1}\right)\right)p_{1}\left(1,t\right)p_{1}\left(2,t\right)t_{\mathrm{passed}} \end{array}$$

Herein, $t_{\mathrm{passed}}=t_{\mathrm{passed}}\left(1\left(t_{0}\right),2\left(t_{0}\right)\right)=t-t_{0}$ is the time passed during the encounter (up to time *t*), which depends on the initial coordinates. While the presentation here is focused on the specific case of pure alignment in two dimensions, the machinery is easily adapted to the case of higher dimensions or more complex interactions. However, the specific expressions as well as the evaluation of the passed time later on are highly dimensionally dependent.

As we consider purely orientational interactions, the dependency on the relative orientation $\Delta =\theta_{2}-\theta_{1}$ will be more critical than that on the relative positions $\Delta x=x_{2}-x_{1}$. Delaying the specifics for a moment, it is helpful to keep track of the proceedings. The result of Equation (13), which was found by means of one-sided molecular chaos considerations to be the solution to Equation (8b), is the missing link to phrase Equation (8a) as a closed evolution equation for the one-particle function $p_{1}$. Specifically, it will be used to evaluate the *Landau* contribution to the collision integral.

### 3.1. Mode Equations

Thus, the relevant equation is the now closed first BBGKY-equation, which we write as

(14) $$\begin{array}{cc} \partial_{t}p_{1}\left(1\right) & =\left(L_{1}+{\mathrm{Pe}}^{\;-1}\partial_{\theta_{1}}^{2}\right)p_{1}\left(1\right)-J_{\mathrm{coll}}^{\mathrm{MF}}\left[p_{1}\right]\left(1\right)-J_{\mathrm{coll}}^{L}\left[p_{1}\right]\left(1\right). \end{array}$$

While we continue to postpone it, evaluation of the collision integrals is possible for a known $p_{1}$. Still, Equation (14) remains an integro-differential equation that is difficult to handle. The strategy that we pursue here is to make use of a Fourier transform. As we are particularly interested in the emergence or relaxation of angular order and generally working in a parameter regime close to the free problem, we only perform this transform with respect to the angular degrees of freedom and assume spatial homogeneity of $p_{1}$ for the rest of this work. This is to keep the presentation concise, and taking the full spatial dependencies into account poses no fundamental problem [15,48,95]. The convention of Fourier transformation we use is that the mode ${\hat{p}}_{m}$ of $p_{1}$ to the wavenumber *m* is given by

(15a) $$\begin{array}{cc} {\hat{p}}_{m}\left(t\right) & =\frac{1}{2\pi }\int_{0}^{2\pi }e^{-im\theta }p_{1}\left(\theta ,t\right)\;\;d\;\theta . \end{array}$$

Consequently, the inverse transformation is given by

(15b) $$\begin{array}{cc} p_{1}\left(\theta ,t\right) & =\sum_{m=-\infty }^{\infty }{\hat{p}}_{m}\left(t\right)e^{im\theta }. \end{array}$$

As the one-particle function is real-valued, we have to have ${\hat{p}}_{m}={\hat{p}}_{-m}^{*}.$

We can generally establish an expectation for the mode equations, i.e., the time evolution of the Fourier modes, which is helpful for guiding one through the proceedings. The kinetic theory we derived is up to the order $O\left(\gamma^{2}\right)∼O\left(M^{2}\right)$, or more physically up to pair terms. Thus, the time evolution of a mode will be a quadratic form in all modes

(16) ∂tp^m=−m2Pe−1p^m+K˜mmvwp^vp^w,

where we directly write the free part of the evolution due to rotational diffusion and use Einstein’s summation convention. We also know that our models are rotationally symmetric: any transformation $\theta_{i}\to \theta_{i}^{\prime }+\alpha$ amounts only to a different choice of reference axis. Therefore, both sides of the mode equations have to display identical behavior under this symmetry operation, making the gauge choice α immaterial. This leads to a sum rule, w+v=m, for the indices in the general approach, which also means that we can reduce the general tensor $\tilde{K}$ to a *mode coupling matrix K*. In summation, we find

(17) $$\begin{array}{cc} \partial_{t}{\hat{p}}_{m} & =-m^{2}{\mathrm{Pe}}^{\;-1}{\hat{p}}_{m}+K_{m}^{w}{\hat{p}}_{w}{\hat{p}}_{m-w}. \end{array}$$

The matrix elements $K_{m}^{w}=V_{m}^{w}+L_{m}^{w}$ each will have contributions $V_{m}^{w}$ from the mean-field or Vlasov description and Landau corrections $L_{m}^{w}$ to it.

#### 3.1.1. The Mean-Field Collision Integral

We recall that the mean-field collision integral was found to be, cp. Equation (9b),

(18) $$\begin{array}{cc} J_{\mathrm{coll}}^{\mathrm{MF}}\left(1\right) & =\gamma \;\partial_{\theta_{1}}\int a_{1,2}\sin\left(n\left(\theta_{2}-\theta_{1}\right)\right)p_{1}\left(\theta_{1}\right)p_{1}\left(\theta_{2}\right)\;\;d\;\theta_{2} \end{array}$$

where we used spatial homogeneity. Inserting Fourier transforms yields

(19) $$\begin{array}{cc} V_{m}^{w}{\hat{p}}_{w}{\hat{p}}_{m-w} & =-\gamma \;\int_{0}^{2\pi }\frac{\;d\;\theta_{1}}{2\pi }e^{-im\theta_{1}}\partial_{\theta_{1}}\int_{0}^{2\pi }\;\;d\;\theta_{2}\sin\left(n\left(\theta_{2}-\theta_{1}\right)\right)\;\;\sum_{k,l=-\infty }^{\infty }\;\;\;\;{\hat{p}}_{k}{\hat{p}}_{l}e^{ik\theta_{1}}e^{il\theta_{2}} \end{array}$$

(20) $$\begin{array}{cc} \; & =-2\pi mi\gamma \sum_{l=-\infty }^{\infty }\int_{0}^{2\pi }\;d\Delta \sin\left(n\Delta \right){\hat{p}}_{m-l}{\hat{p}}_{l}e^{il\Delta } \end{array}$$

by integration by parts and changing integration variables. Inspecting the interaction kernel $\sin\left(n\Delta \right)=\frac{1}{2i}\left(e^{in\Delta }-e^{-in\Delta }\right)$, performing the integral is trivial, and we can directly infer that

(21) $$\begin{array}{cc} V_{m}^{w} & =m\pi \;\gamma (\delta_{n}^{w}-\delta_{-n}^{w}) \end{array}$$

Two conclusions are immediate from this result. One, the modes selected by the global chelation number *n* couple to the conserved mode ${\hat{p}}_{0}$. Thus, they will be dominant hydrodynamically, which was to be expected. Secondly, there is an implied timescale $\tau_{n}^{-1}=n\pi \gamma$ for the relaxation dynamics of this dominant mode. This is also to be expected, as it reflects the perturbative behavior $\sin(n\left(\Delta_{0}+\varepsilon \right))\approx n\varepsilon$ around the equilibrium positions.

The mean-field description should intuitively be the correct description for M≫1, as there will be no relevant correlations in the fully-connected model. This *propagation of chaos* property is indeed correct [35,55]. However, this limit should be approached carefully in this model. Inspection of the interaction in Equation (1) shows that M→∞ (i.e., R→∞ and effectively crossing over into a moving, fully-connected XY-model) is only sensible if we rescale Γ→Γ/M, i.e., Sc→Sc/M. We find corrections to the mean-field of order $M{\mathrm{Sc}}^{2}$, which are therefore negligible in this limit, validating earlier propagation of chaos results within our Landau approach.

#### 3.1.2. The Landau Collision Integral

Collecting results, we can write the Landau collision integral as

(22) $$\begin{array}{cc} J_{\mathrm{coll}}^{L}\left(1,t\right) & =M{\mathrm{Sc}}^{2}\;\partial_{\theta_{1}}\;\;\;\int \int \;\;\;\sin\left(n\Delta \right)t_{\mathrm{passed}}\;\left(\partial_{\theta_{1}}-\partial_{\theta_{2}}\right)\sin\left(n\Delta \right)p_{1}p_{1}\left(2,t\right)\;\;d\;x_{2}\;d\theta_{2}. \end{array}$$

In this case, we cannot directly proceed by evaluating the spatial integration, as the passed time $t_{\mathrm{passed}}$ depends on it. We recall that this is meant to be the time an encounter lasted up to time *t* that started at $t_{0}$ with particles coordinates at time $t_{0}$ given by 1 and 2, respectively. Encounters have to end at a relative distance given by the interaction radius, $|x_{2}-x_{1}|=R$. Choosing a frame of reference such that $x_{2}\left(t_{0}\right)=x_{1}\left(t_{0}\right)+{\left(-\cos\phi ,\sin\phi \right)}^{T}$, with ϕ being the angle relative to the relative velocity $v_{\mathrm{rel}}=v_{2}-v_{1}=\left({\hat{n}}_{2}-{\hat{n}}_{1}\right)$ whose modulus computes to $v_{\mathrm{rel}}=2\left|\sin\frac{\Delta }{2}\right|$, we find from geometry that the distance traversed at time *t* is

(23) $$\begin{array}{cc} s(t,t_{0}) & =x\left(t\right)+\cos\phi \left(t_{0}\right). \end{array}$$

wherein cosϕ≥x(t)≥−cosϕ≥0. We have to consider any possibility for $t_{0}$, which simply means that $x_{2}$ is uniformly distributed within the interaction radius around $x_{1}$ at time *t*. Thus, we can compute the *spatially averaged* passed time

(24) $$\begin{array}{cc} t_{⊙} & =\frac{2}{\pi }\int_{0}^{1}\;d\;y\int_{0}^{\sqrt{1-y^{2}}}\;\;\;\;\;\;\;\;\;\;\;\;d\;x\;\;\;\;\frac{x+\sqrt{1-y^{2}}}{v_{\mathrm{rel}}}=\frac{4}{\pi v_{\mathrm{rel}}}\int_{0}^{1}\;d\;y\;\left(1-y^{2}\right)=\frac{8}{3\pi v_{\mathrm{rel}}} \end{array}$$

Plugging the averaged passed time into the Landau collision integral yields

(25) $$\begin{array}{cc} J_{\mathrm{coll}}^{L}\left(\theta_{1},t\right) & =M{\mathrm{Sc}}^{2}\partial_{\theta_{1}}\;\;\int \;\;\sin\left(n\Delta \right)\pi t_{⊙}\;\left(\partial_{\theta_{1}}-\partial_{\theta_{2}}\right)\sin\left(n\Delta \right)p_{1}\left(\theta_{1}\right)p_{1}\left(\theta_{2}\right)\;d\theta_{2}\\ \; & =\frac{4}{3}M{\mathrm{Sc}}^{2}\partial_{\theta_{1}}\;\;\int \;\;\frac{\sin\left(n\Delta \right)}{\left|\sin\frac{\Delta }{2}\right|}\;\left(\partial_{\theta_{1}}-\partial_{\theta_{2}}\right)\sin\left(n\Delta \right)p_{1}\left(\theta_{1}\right)p_{1}\left(\theta_{2}\right)\;d\theta_{2}. \end{array}$$

Thus, we receive after Fourier transformation

(26) $$\begin{array}{cc} \; & L_{m}^{w}{\hat{p}}_{w}{\hat{p}}_{m-w}\;\;=\;\frac{4miM{\mathrm{Sc}}^{2}}{3}\;\;\;\;\;\sum_{v=-\infty }^{\infty }\;\;\;\;{\hat{p}}_{m-v}{\hat{p}}_{v}\;\;\int_{0}^{2\pi }\;\;\;\;\;\;\;d\;\Delta \frac{\sin\;(n\Delta )}{\left|\sin\frac{\Delta }{2}\right|}\left(\;-2n\;\cos\;(n\Delta )+i\left(m-2v\right)\;\sin\;(n\Delta )\right)e^{il\Delta } \end{array}$$

(27) $$\begin{array}{cc} \; & \;=\;\frac{4mM{\mathrm{Sc}}^{2}}{3}\;\;\;\;\sum_{v=-\infty }^{\infty }\;\;\;\;{\hat{p}}_{m-v}{\hat{p}}_{v}\;\;\int_{0}^{2\pi }\;\;\;\;\;\;\;d\;\Delta \frac{\sin\left(n\Delta \right)}{\left|\sin\frac{\Delta }{2}\right|}\left(2n\cos\;(n\Delta )\sin\;(v\Delta )-\left(m-2v\right)\sin\;(n\Delta )\cos\;(v\Delta )\right) \end{array}$$

We outline the calculation of this ultimately trivial integral for the case of general *n* and, specifically, perform the calculation for n=1,2 in the Appendix A. After collecting terms, we end up at

(28) $$\begin{array}{cc} L_{m}^{w}{|}_{n=1}= & mM{\mathrm{Sc}}^{2}\frac{64}{3}\frac{\left(\left(4w^{2}-3\right)m-4w\right)}{\left(2w-1)(2w+1)(2w-3)(2w+3\right)} \end{array}$$

(29) $$\begin{array}{cc} L_{m}^{w}{|}_{n=2}= & mM{\mathrm{Sc}}^{2}\frac{256}{3}\frac{\left(\left(64w^{6}-848w^{4}+2524w^{2}-1155\right)m-320w^{5}+2848w^{3}-4148w\right)}{\left(2w-1)(2w+1)(2w-3)(2w+3)(2w-5)(2w+5)(2w-7)(2w+7\right)}. \end{array}$$

**Figure 5:**
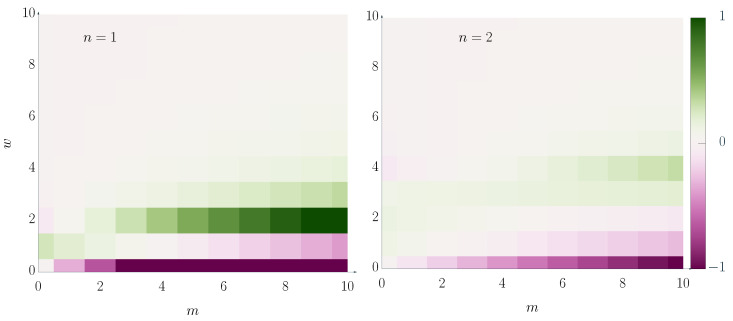
Graphical representation of the Landau contributions, $L_{m}^{w}$, to the collisional mode coupling matrix. We omit constant pre-factors and some values are outside the value range depicted here to increase clarity. We find that these second-order corrections display a clear self-mixing ($L_{0}^{w}$ is negative).

We show a graphical representation of these Landau contributions to the mode coupling matrix in Figure 5. The manifestation of the symmetry imposed onto the system via the chelation number *n* is less intuitive than for the mean-field contributions that connect the chelation mode to the density. In the second order, the structure of the corrections, $K_{m}^{w}{\hat{p}}_{m-w}{\hat{p}}_{w}$, means that there are two elements that contribute to the coupling of the *m*th mode to the density, $K_{m}^{0}$ and $K_{m}^{m}$. The second order part of the former is strongly negative, while that of the latter is weakly positive. Their sum, however, is negative, meaning that taking into account second-order contributions has (for n=2 and n=1) an effect that counteracts *spontaneous alignment*.

The coupling matrix elements $K_{m}^{w}$, or rather the full mode equations of Equation (17), contain the full dynamical information within the made assumptions (spatial homogeneity, cutoff at order $\gamma^{2}$). As such, they can be used to infer predictions on many relevant quantities. For example, we can consider the hydrodynamic expansion around an unordered state that we alluded to earlier, i.e., we assume that all $p_{m}$ for m>0 are small quantities. The zeroth mode $p_{0}=\frac{1}{2}$ corresponds to the density and is a conserved quantity. This yields

(30) $$\begin{array}{c} \partial_{t}{\hat{p}}_{m}\approx -m^{2}{\mathrm{Pe}}^{\;-1}{\hat{p}}_{m}+\frac{1}{2}\;\left(K_{m}^{m}+K_{0}^{m}\right){\hat{p}}_{m}. \end{array}$$

For the mode that is selected by the symmetry encoded into the chelation number *n*, this means

(31) $$\begin{array}{c} \partial_{t}{\hat{p}}_{n}\approx -n^{2}{\mathrm{Pe}}^{\;-1}{\hat{p}}_{n}+\gamma \;\alpha_{n}-\gamma^{2}\;\beta_{n} \end{array}$$

Here, we separate off the γ-dependencies from the Vlasov (α>0) and Landau (β>0) contributions. Thus, it is directly evident that the onset of instability of this unordered state shifts towards *lower* values of ${\mathrm{Pe}}^{\;-1}$, i.e., lower noise.

## 4. Comparison with Agent-Based Simulation

The big advantage of kinetic theory based on the microscopic equations of motion versus more global hydrodynamic approaches is that we can validate our findings directly by comparison to agent-based simulation without any free parameters. Using an Euler scheme to numerically integrate Equation (1) [96,97,98], we measure the Fourier modes. This is done for the deterministic system (${\mathrm{Pe}}^{\;-1}=0$), starting from a polarized, spatially uniform state—see Figure 3 for some exemplary snapshots. Relaxation from an ordered state for γ<0 is a direct way of assessing the correctness of the angular mode couplings, as there will be no spatial patterns emerging (as would be the case for γ>0 or with strong noise). In particular, we initialize the directions uniformly θ∈[−α/2,α/2] with α=5π/6. A secondary benefit of this choice is that the system is polarized along the *x*-axis, which in our convention means that ensemble-averaged Fourier modes will always be real-valued functions.

**Figure 6:**
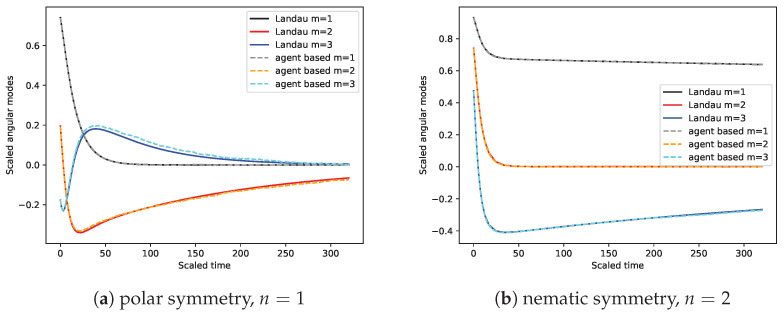
Fourier modes for systems at ${\mathrm{Pe}}^{\;-1}=0$ with n=1 and for n=2. The modes extracted from the agent-based simulation (dashed lines, averaged over $N_{\mathrm{sample}}=80$ realizations) are compared to the theoretically predicted modes (labeled Landau, solid lines) derived by the Landau equation kinetic theory approach. The scaled time is $t^{\prime }=\mathrm{Sc}t$. It is qualitatively intuitive that lower values of the coupling constant Γ are necessary to see similarly small deviations in the n=2 case as in the n=1 case, because linearization of the interaction kernel, cp. Equation (1), around equilibria yields a response $\dot{\delta \theta }\approx -n\Gamma \delta \theta$ to a deviation δθ. The parameters used include the following: M=0.1, N=493, R=1, $v_{0}=4$, Δt=0.0025 and (**a**) Γ=−0.2, $MSc^{2}=2.5\cdot 10^{-4}$, n=1, (**b**) Γ=−0.02, $MSc^{2}=2.5\cdot 10^{-6}$, n=2.

In Figure 6, we show that there is quantitative agreement between the measured mode dynamics in agent-based simulation and the results obtained from numerical integration of the mode equations, cp. Equation (17). The mode equations are numerically integrated by setting $p_{m}=0$ for all |m|>10. Here, we use rather small system sizes of N=493, but we do not expect qualitative differences with respect to the mode dynamics. For larger systems, spatial instabilities can become relevant, making the assumption of spatial homogeneity false [18,99,100]. However, omitting the spatial derivatives was done to keep the presentation concise, and fully accounting for them is not a principle difficulty. Thus, there are relevant contributions from terms beyond the mean-field at any *N* [42,50,101]. As discussed before, this is the result of fixing *M* and Sc to finite values as control parameters for any *N*. If the limit N,M→∞, Sc→0 with MSc fixed were to be considered instead, then we would expect the corrections to the mean-field to vanish for large *N*, establishing the propagation of chaos [35,55].

An important transport quantity that is suited to determination within this approach is the self-diffusion constant $D_{\mathrm{self}}$ at negative coupling when the system is self-mixing. Labeling a pivot particle with index 1, we define it as

(32) $$\begin{array}{cc} D_{\mathrm{self}} & =\lim_{t,\tau \to \infty }\frac{1}{\tau }\langle {\left(x_{1}\left(t+\tau \right)-x_{1}\left(t\right)\right)}^{2}\rangle \end{array}$$

By means of a Green–Kubo relation, this is directly linked to the velocity correlation time $\tau_{c}$, which determines the decay of the velocity autocorrelation function $\langle v\left(t+\tau \right)\cdot v\left(t\right)\rangle ∼e^{-\tau /\tau_{c}}$ as $\tau_{c}=2D_{\mathrm{self}}$. As has been pointed out before in ref. [15], this quantity does not exist in mean-field calculations. The calculation via a Boltzmann–Lorentz-type theory is easily adapted, and we do not reproduce it in full here, but a rough argument would be as follows: if we label a very small fraction as tracer particles with modes ${\hat{q}}_{m}$, then their interaction partners will essentially always be with regular particles whose modes we denote by ${\hat{p}}_{m}$. Going through the derivation of Equation (17), it is apparent that the analogue for modes of the tracer particles, which explicitly takes into account spatial derivatives as is necessary for diffusive phenomena, would be

(33) $$\begin{array}{cc} \partial_{t}{\hat{q}}_{m}+\frac{1}{2}\left(\nabla^{-}{\hat{q}}_{m-1}+\nabla^{+}{\hat{q}}_{m+1}\right) & =-m^{2}{\mathrm{Pe}}^{\;-1}{\hat{q}}_{m}+K_{m}^{w}{\hat{q}}_{m-w}{\hat{p}}_{w} \end{array}$$

with the shorthand $\nabla^{\pm }=\partial_{x}\pm i\partial_{y}$. Again taking the limit of an unordered background, ${\hat{p}}_{m}\propto \delta_{m,0}$, we see that the mean velocity of the tracer particles, ${\hat{q}}_{1}$, only couples with the background density via $K_{1}^{0}$, an element that does not exist in the mean-field irrespective of *n*. In particular, we find a decay of the velocity autocorrelation function consistent with

(34) $$\begin{array}{cc} D_{\mathrm{self}} & =-\frac{1}{K_{1}^{0}-{\mathrm{Pe}}^{-1}}. \end{array}$$

**Figure 7:**
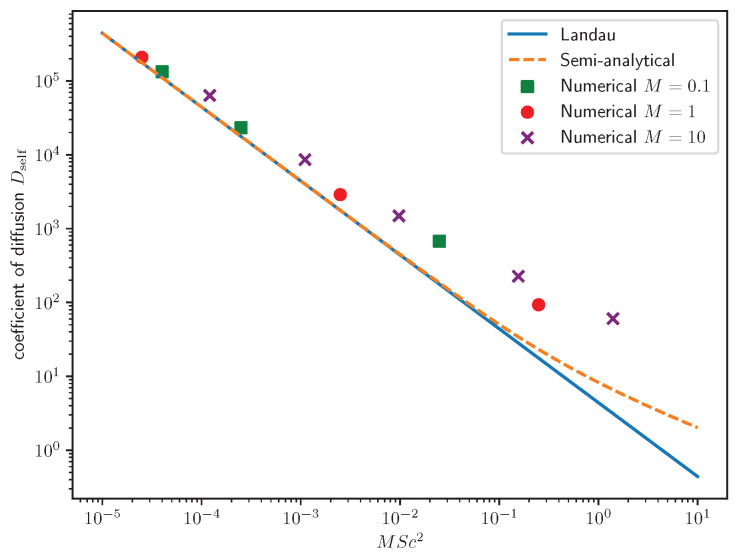
Self-diffusion coefficient $D_{\mathrm{self}}$ as a function of the scale of corrections beyond mean-field theory. We present numerical results (parameters as in Figure 6, squares for M=0.1, circles for M=1, saltires for M=10) from agent-based simulations, which are to be compared with the prediction (no free parameters) of Equation (34) (solid blue line). Improving the effective random-telegraph description (dashed orange line), especially finding better approximations than assuming the independence of spatial and angular variables, is the subject of future research—see the Discussion in the main text and Appendix B.

We present numerical results for $D_{\mathrm{self}}$ as inferred from the velocity auto-correlation function in Figure 7. The data are found in quantitative agreement for sufficiently small values of $M{\mathrm{Sc}}^{2}$, which is the order first correction beyond mean-field theory. Similar to the discussion of the polar case [15,48], the scaling itself (and thus the validity of only considering Landau equation contributions) and, at least qualitatively, corrections to scaling can be understood by a random telegraph model (labeled semi-analytical in Figure 7), which we reproduce in the Appendix B. Interestingly and not explicitly captured in the telegraph process description, the diffusion coefficients’ dependency on the chelation number *n* is non-trivial. At ${\mathrm{Pe}}^{\;-1}=0$, we find $D_{\mathrm{self}}^{n=2}/D_{\mathrm{self}}^{n=1}=\frac{35}{44}\approx 0.8$. This is indicative of self-diffusion being a result of the full-interaction kernel, whereas other properties could be understood from the perspective of linearization around equilibria leading to power-law scaling in *n*.

## 5. Discussion

In summation, we gave a concise introduction into Landau equation-based methods of kinetic theory, as they can be applied to active matter with few prerequisites. This was performed for a specific Vicsek-like model with interactions of *n*-fold symmetry. By means of careful inspection of the relevant scalings, it becomes apparent that the Landau equation is not the mere weak coupling limit of the Boltzmann equation, but rather a separate approach to finding a solution to the BBGKY hierarchy. The Boltzmann equation is fundamentally an expansion in the density, whereas the Landau equation is an expansion in the coupling strength, valid at any density (as long as the effective interactions MSc are sufficiently small).

We derived the full mode equations, establishing the connection between the mean-field (or Vlasov or McKean) and the Landau contributions to the assumptions of molecular chaos and the more physical assumption of one-sided molecular chaos, respectively. The former neglects all inter-particle correlations, whereas the latter takes correlations *during* interactions into account. The main technical results are the elements beyond the mean-field of the mode coupling matrix (see Figure 5).

As a result of this, we are able to analytically determine an asymptotic expression for the self-diffusion coefficient of the self-propelled particles, a measure for the self-mixing they display at anti-aligning couplings even in a deterministic system. This result is then independently corroborated by a random-telegraph process that gives identical asymptotics and offers some insight into corrections to scaling at larger couplings. As such, we give an example demonstrating that first-principle kinetic theories for active matter, when properly derived from the exact Liouville– or Fokker–Planck equation using the weaker, generic assumption of one-sided molecular chaos, lead to quantitative agreement, even in transient/dynamic situations far from stationary states. This is easily underappreciated in favor of more qualitative descriptions based on strong, problem-specific assumptions [8].

We hope that this work serves as one element refamiliarizing a broader community with the ideas and merits of approaching statistical physics kinetically starting from microscopic equations of motion. Going forward, these equations will provide a valuable set of tools in the understanding of active matter.

## Data Availability

Data and source code available upon request from the authors.

## Appendix A. Evaluation of Collision Integrals

In this section, we give some purely algebraic details on the actual evaluation of the collision integrals. As we will show, such an evaluation is possible for an arbitrary integer *n*. However, we did not succeed in finding a succinct closed expression that holds for any *n*, so we will limit the specifics to the most relevant cases of n=1 and n=2.

The evaluation of the Landau collision integral reduces to computing two families $A_{n,v}$, $B_{n,v}$ of integrals

(A1a) $$\begin{array}{cc} A_{n,v} & =\int_{-\pi }^{\pi }\;\;\;d\;\Delta \frac{\sin\left(n\Delta \right)}{\left|\sin\left(\frac{\Delta }{2}\right)\right|}\cos\left(n\Delta \right)\sin\left(v\Delta \right) \end{array}$$

(A1b) $$\begin{array}{cc} B_{n,v} & =\int_{-\pi }^{\pi }\;\;\;d\;\Delta \frac{\sin\left(n\Delta \right)}{\left|\sin\left(\frac{\Delta }{2}\right)\right|}\sin\left(n\Delta \right)\cos\left(v\Delta \right). \end{array}$$

We adapt textbook trigonometric formulae [102] to find

(A2a) sin(nΔ)=sinΔ2∑k=0⌊2n−12⌋(−1)k2n−k−1k22n−2k−1cos2n−2k−1Δ2

(A2b) cos2n−2k−1Δ2=41−n+k∑l=0n−k−12n−2k−1lcos((2n−2k−1−2l)Δ2)

(A2c) sin(nΔ)sinΔ2=∑k=0⌊2n−12⌋(−1)k2n−k−1k2∑l=0n−k−12n−2k−1lcos((2n−2k−1−2l)Δ2)

(A2d) $$\begin{array}{cc} \sin\left(n\Delta \right)\cos\left(v\Delta \right) & =\frac{1}{2}\left(\sin\left(\left(n-v\right)\Delta \right)+\sin\left(\left(n+v\right)\Delta \right)\right) \end{array}$$

Thus, it is evident that the computation of $A_{n,v},B_{n,v}$ can be reduced to integrals of the form ∫dΔf(αΔ)g(βΔ), with f,g being sine or cosine and α+1/2,β∈Z.

We do not attempt to evaluate this in general, but give specific results for the commonly studied polar case, n=1,

(A3) $$\begin{array}{cc} A_{1,v}^{n=1} & =2\;\int_{0}^{\pi }\;\;\;d\;\Delta \;\frac{\sin(\Delta )}{\sin\frac{\Delta }{2}}\cos\Delta \sin\left(v\Delta \right)=2\int_{0}^{\pi }\;\;\;d\;\Delta \;\cos\frac{\Delta }{2}\left(\sin\left(\left(v-1\right)\Delta \right)+\sin\left(\left(v+1\right)\Delta \right)\right)\\ \; & \int_{0}^{\pi }\;\;\;d\;\Delta \;\cos\frac{\Delta }{2}\sin\left(w\Delta \right)=\frac{1}{4i}\int_{0}^{\pi }\;\;\;d\;\Delta \;\left(e^{i\Delta /2}+e^{-i\Delta /2}\right)\left(e^{iw\Delta }-e^{-iw\Delta }\right)\overset{w\in Z}{=}\frac{1}{w^{2}-1/4} \end{array}$$

(A4) $$\begin{array}{cc} A_{1,v}^{n=1} & =2\left(\frac{1}{{\left(v-1\right)}^{2}-1/4}+\frac{1}{{\left(v+1\right)}^{2}-1/4}\right)=B_{1,v}^{n=1} \end{array}$$

and the nematic case, n=2

(A5) $$\begin{array}{cc} A_{1,v}^{n=2} & =2\;\int_{0}^{\pi }\;\;\;d\;\Delta \;\frac{\sin(2\Delta )}{\sin\frac{\Delta }{2}}\cos\left(2\Delta \right)\sin\left(v\Delta \right) \end{array}$$

(A6) $$\begin{array}{cc} & =4\int_{0}^{\pi }\;\;\;d\;\Delta \;\cos\frac{\Delta }{2}\cos\Delta \left(\sin\left(\left(v-2\right)\Delta \right)+\sin\left(\left(v+2\right)\Delta \right)\right) \end{array}$$

(A7) $$\begin{array}{cc} \; & =2\int_{0}^{\pi }\;\;\;d\;\Delta \;\cos\frac{\Delta }{2}\left(\sin\left(\left(v-3\right)\Delta \right)+\sin\left(\left(v-1\right)\Delta \right)+\sin\left(\left(v+1\right)\Delta \right)+\sin\left(\left(v+3\right)\Delta \right)\right) \end{array}$$

(A8) $$\begin{array}{cc} \; & =2\left(\frac{1}{{\left(v-3\right)}^{2}-1/4}+\frac{1}{{\left(v-1\right)}^{2}-1/4}+\frac{1}{{\left(v+1\right)}^{2}-1/4}+\frac{1}{{\left(v+3\right)}^{2}-1/4}\right)=B_{1,v} \end{array}$$

## Appendix B. Self-Diffusion by Means of a Random Telegraph Model

In this section, we present a different approach [15,48] to computing the self-diffusion constant based on the notion that changes in angle and changes in adjacency effectively decouple for Γ<0, M≫1.

We recall that the angular dynamics are given by, cp. Equation (1b),

$$\begin{array}{cc} \dot{\theta_{i}} & =\Gamma \;\sum_{j\in \Omega_{i}}\sin\left(n\left(\theta_{j}\left(t\right)-\theta_{i}\left(t\right)\right)\right)+\eta_{i} \end{array}$$

and notice that particularly in the case of many neighbors, $|\Omega_{i}|∼M\gg 1$, both contributions are of a similar noisy character (as we consider Γ<0 and thus expect limited angular correlations). This leads us to tentatively write

(A9) $$\begin{array}{cc} \dot{\theta_{i}} & =\xi_{i}+\eta_{i} \end{array}$$

with an *intrinsic noise* $\xi_{i}$ whose properties are to be determined. From this, we directly conclude that the angular diffusion, which we can use to measure the velocity decorrelation since $v\left(\theta +\Delta \theta \right)\cdot v\left(\theta \right)=\cos\left(\Delta \theta \right)\approx 1-{\left(\Delta \theta \right)}^{2}/2$, is given by

(A10) $$\begin{array}{cc} \langle \Delta \theta^{2}\rangle \; & =\;\int_{t}^{t+\tau }\;\;\;\int_{t}^{t+\tau }\left(\langle \xi_{i}\left(\tilde{t}\right)\xi_{i}\left(t^{\prime }\right)\rangle +\langle \eta_{i}\left(\tilde{t}\right)\eta_{i}\left(t^{\prime }\right)\rangle +\langle \xi_{i}\left(t^{\prime }\right)\eta_{i}\left(\tilde{t}\right)\rangle +\langle \xi_{i}\left(\tilde{t}\right)\eta_{i}\left(t^{\prime }\right)\rangle \right)\;d\;\tilde{t}\;d\;t^{\prime }. \end{array}$$

The second term is known, and the last two, at first approximation, do not contribute, since the two noise terms can be taken as independent. To compute the first term, we again introduce indicator functions $a_{i,j}$, such that $\sum_{j\in \Omega_{i}}=\sum_{j}a_{i,j}$. These are again well approximated as being independent of the angular degrees of freedom, and we introduce a time-correlator

(A11) $$\begin{array}{c} \langle a_{i,j}\left(\tilde{t}\right)a_{i,k}\left(t^{\prime }\right)\rangle =\delta_{j,k}g\left(\tilde{t}-t^{\prime }\right)\;\mathrm{for}\;i\neq j,k. \end{array}$$

That is, we model the indicator functions as the results of a random telegraph process [82], a signal that is either zero or one, happening independent of the angular dynamics. This is a choice and somewhat obviously incorrect in a strict sense, as the indicator functions act on the spatial coordinates, which are given by integrated velocities and therefore depend on the entire history of all angles. In actuality, the beginning and endings of interactions in a dense state of essentially randomly oriented agents will be almost independent of the respective angles. Changes in adjacency will happen on some typical timescale that we characterize by an off-rate $\omega_{\mathrm{off}}$, and choosing pre-factors to be convenient later on, we write

(A12) $$\begin{array}{c} \lim_{N\to \infty }\left(N-1\right)g\left(\tilde{t}-t^{\prime }\right)=Me^{-\omega_{\mathrm{off}}\left|\tilde{t}-t^{\prime }\right|} \end{array}$$

Computing the angular expressions as well [15], we find a *self-consistent* equation for $\Delta \theta^{2}$

(A13) $$\begin{array}{c} \langle \Delta \theta^{2}\rangle =\Gamma^{2}M\int_{0}^{\tau }dt\left(\tau -t\right)e^{-\omega_{\mathrm{off}}t-n^{2}\langle \Delta \theta_{1}^{2}\rangle }+D_{r}\tau \end{array}$$

which can be solved by rephrasing it as a differential equation (taking derivatives with respect to τ twice), resulting in

(A14) $$\begin{array}{cc} \langle \Delta \theta^{2}\rangle & =\frac{1}{n^{2}}\left(2\ln\frac{1+e^{2|t|a\sqrt{\frac{\Gamma^{2}n^{2}M}{2}}}c^{2}}{2e^{|t|a\sqrt{\frac{\Gamma^{2}n^{2}M}{2}}}ca}-\omega_{\mathrm{off}}\left|t\right|\right). \end{array}$$

with $c=a+\sqrt{a^{2}-1}$ and $a=\sqrt{\frac{{\left(n^{2}D_{r}+\omega_{\mathrm{off}}\right)}^{2}}{2M\Gamma^{2}n^{2}}+1}$. Inspecting these results for large timescales, $t\gg \omega_{\mathrm{off}}^{-1}$, we find an effective angular noise

(A15) $$\begin{array}{c} \tilde{D_{r}}=\frac{\omega_{\mathrm{off}}}{n^{2}}\left(\sqrt{\frac{W}{\omega_{\mathrm{off}}^{2}}+\frac{2M\Gamma^{2}n^{2}}{\omega_{\mathrm{off}}^{2}}}-1\right) \end{array}$$

with $W={\left(n^{2}D_{r}+\omega_{\mathrm{off}}\right)}^{2}$. For an easier comparison, we perform the same non-dimensionalization as in the main text and, using λ as the non-dimensional off-rate, find

(A16) $$\begin{array}{cc} D_{\mathrm{self},\mathrm{tele}}^{-1} & =\frac{\lambda }{n^{2}}\left(\sqrt{\frac{{\left(2n^{2}Pe^{-1}+\lambda \right)}^{2}}{\lambda^{2}}+\frac{2M{\mathrm{Sc}}^{2}n^{2}}{\lambda^{2}}}-1\right) \end{array}$$

Inspecting this in the relevant limit, ${\mathrm{Pe}}^{-1}=0$, $M{\mathrm{Sc}}^{2}\ll 1$, we find that the telegraph process predicts the same asymptotics as the Landau equation approach, cp. Equation (34),

(A17) $$\begin{array}{cc} D_{\mathrm{self},\mathrm{tele}} & \approx \lambda^{-1}M{\mathrm{Sc}}^{2}. \end{array}$$

One way of determining λ would be via a measurement of first-passage times. However, we can also demand the two approaches to coincide in the limit $M{\mathrm{Sc}}^{2}\ll 1$. This therefore gives the semi-analytical curve shown in Figure 7.

We also take note of the fact that the final result for ${\mathrm{Pe}}^{-1}=0$ has no explicit dependence on the chelation number *n*. This can be understood by going back to the angular equation of motion Equation (1b). Multiplying this equation by *n* rescaling $n\Gamma =\Gamma^{*}$, we can eliminate any *n*-dependence in the deterministic parts by considering $\theta^{*}=n\theta$. Thus, the angular diffusion in the small ${\mathrm{Pe}}^{-1}$-limit, $\Delta {\left(\theta^{*}\right)}^{2}\propto {\left(\Gamma^{*}\right)}^{2}$, will also show no explicit *n*-dependence. This rescaling does not work for the spatial dynamics, as the propulsion is not *n*-dependent. Therefore, the (here neglected) coupling of spatial and angular degrees of freedom has to induce a (weak) dependence on *n*. This is covered by the Landau approach.
